# Marker Genes Change of Synovial Fibroblasts in Rheumatoid Arthritis Patients

**DOI:** 10.1155/2021/5544264

**Published:** 2021-06-04

**Authors:** Lifen Liao, Ke Liang, Lan Lan, Jinheng Wang, Jun Guo

**Affiliations:** ^1^Department of Laboratory, Affiliated Hospital of Guilin Medical University, Xiufeng District, Guilin, 541001 Guangxi, China; ^2^Department of Laboratory, Nanxishan Hospital of Guangxi Zhuang Autonomous Region, Xiangshan District, Guilin, 541002 Guangxi, China; ^3^Department of Hematology, Rizhao People's Hospital, Donggang District, Rizhao, 276800 Shandong, China

## Abstract

**Background:**

Rheumatoid arthritis (RA) is a chronic condition that manifests as inflammation of synovial joints, leading to joint destruction and deformity.

**Methods:**

We identified single-cell RNA-seq data of synovial fibroblasts from RA and osteoarthritis (OA) patients in GSE109449 dataset. RA- and OA-specific cellular subpopulations were identified, and enrichment analysis was performed. Further, key genes for RA and OA were obtained by combined analysis with differentially expressed genes (DEGs) between RA and OA in GSE56409 dataset. The diagnostic role of key genes for RA was predicted using receiver operating characteristic (ROC) curve. Finally, we identified differences in immune cell infiltration between RA and OA patients, and utilized flow cytometry, qRT-PCR, and Western blot were used to examine the immune cell and key genes in RA patients.

**Results:**

The cluster 0 matched OA and cluster 3 matched RA and significantly enriched for neutrophil-mediated immunity and ECM receptor interaction, respectively. We identified 478 DEGs. In the top 20 degrees of connection in the PPI network, the key genes for RA were obtained by comparing with the gene markers of cluster 0 and cluster 3, respectively. ROC curve showed that CCL2 and MMP13 might be diagnostic markers for RA. We found aberrant levels of CD8+T, neutrophil, and B cells in RA fibroblasts, which were validated in clinical samples. Importantly, we also validated the differential expression of key genes between RA and OA.

**Conclusion:**

High expression of CCL2 and MMP13 in RA may be a diagnostic and therapeutic target.

## 1. Introduction

Rheumatoid arthritis (RA) is a chronic inflammatory disease that affects joint synovial tissue, causing joint pain and disability [1]. RA is characterized by infiltration of synovium with inflammatory cells, hyperplasia of synovial fibroblasts, and progressive inflammation of the joint, leading to cartilage destruction, bone erosion, and disability [2, 3]. A large population studies found that in RA patients, the mortality was increased with years compared to the general population [4]. Within 10 years of RA onset, at least 50% of patients in developed countries are unable to take full-time jobs, probably due to the resulting disability [5]. Patients with RA represent approximately 0.5%-1% of the world's population and have regional variations [6]. The prevalence is higher in women aged between 35 and 50 years than in age-matched men [7].

The etiology of RA is complex and includes not only genetic and epigenetic factors but also smoking, infection, microbiota, and others [8]. Although the etiology of RA is not fully understood, its autoimmune properties have been widely recognized. Autoreactive CD4+T cells will stimulate macrophages and synovial fibroblasts to secrete cytokines, including TNF, IL-1, and IL-17, which contribute to invasive vasculitis through recruitment of immune cells and expansion of synovial fibroblasts [9, 10]. In addition, RA has a specific tissue response characterized by an aggressive proinflammatory phenotype of local fibroblasts with stromal regulatory, osteoclastogenic, and invasive properties [11]. In RA, stable reprogramming of synovial fibroblasts disrupts their protective regulatory processes, promotes their survival, and increases their production of proinflammatory agents and proteases [12]. Rheumatoid synovial fibroblasts are able to generate and support a sustained leukocyte infiltration [13]. Therefore, a deep and extensive understanding of synovial fibroblasts and their potential roles in the pathogenesis of RA is of great importance for the prevention and treatment of RA.

With the continuous development of medical standards, conventional treatments can alleviate the condition of RA patients, but cannot completely cure them [14]. Notably, patient awareness of RA, the willingness to seek medical treatment, the time to receive appropriate treatment, and the diagnostic capability of physicians all influence the treatment and prognosis of RA [15]. Early diagnosis and treatment often delay and prevent joint deformities, improve the quality and life span of patients, and are a prerequisite for early detection of patients.

Therefore, it is very important to explore the pathogenesis of RA to develop accurate treatments and new drug targets. This study broadens the candidate list of therapeutic targets for RA, as well as the underlying mechanisms of inflammatory infiltration, by exploring the differences in gene expression and the different biological functions between RA and OA patients. It was further clarified that chemokines and matrix metalloproteinases (MMPs) signaling were involved in the pathological process of RA associated with immune cell infiltration.

## 2. Materials and Methods

### 2.1. Rheumatoid Arthritis Data Collection

Data used in this study were obtained from the gene expression omnibus (GEO) public database (https://www.ncbi.nlm.nih.gov/geo). GSE109449 included gene expression profile of 192 single synovial fibroblasts from 2 rheumatoid arthritis (RA) patients and 192 single synovial fibroblasts from 2 osteoarthritis (OA) patients through single-cell RNA-seq (scRNA-seq) based on GPL18573 [16].

GSE56409 included gene expression profile of fibroblasts which were isolated from synovium, bone marrow, or skin tissue samples of 12 rheumatoid arthritis patients and 6 osteoarthritis patients at the time of knee or hip replacement surgery based on GPL570 of array [17]. The raw data was processed and normalized using the Robust Multiarray Averaging (RMA) methodology.

### 2.2. Processing of the scRNA-Seq Data

The quality control, statistical analysis, and exploration of the scRNA-seq data for GSE109449 were performed using the Seurat R package [18, 19]. Principal component analysis (PCA) was used to identify significantly available dimensions with a *P* value < 0.05. The uniform manifold approximation and projection (UMAP) algorithm [20] was used for the visualization of unsupervised clustering. The differential expression analysis was used the limma R package [21, 22]. Set the filtering threshold *P* value < 0.05. Different cell clusters were annotated by the singleR package [23].

### 2.3. Enrichment Analysis

Biological process (BP) in Gene Ontology (GO) and Kyoto Encyclopedia of Genes and Genomes (KEGG) enrichment analysis [24–27] of gene markers of clusters was performed using Enrichr online tool [28]. The *P* value < 0.05 was considered significantly enriched.

### 2.4. Difference Analysis

Differential expression analysis between RA and OA in GSE56409 was performed using the limma R package [21, 29]. Genes with an |log2(FoldChange)| >1 and *P* value < 0.05 were identified as differentially expressed genes (DEGs).

### 2.5. Generation of Protein-Protein Interaction (PPI) Network

The PPI network of common genes was identified through the Search Tool for the Retrieval of Interacting Genes (STRING) (http://string-db.org/) database. The combined score >0.7 was considered significant. The PPI network was visualized by the Gephi software [30]. The PPI network genes were ranked based on their degree of connectivity with other genes.

### 2.6. Identification of Immune Cell Infiltration

The marker gene sets of different immune cell types were obtained from Bindea et al [31]. We used the single-sample gene set enrichment analysis (ssGSEA) in R package GSVA [32] to derive the enrichment scores of each immune cell. The ssGSEA applies gene signatures expressed by immune cell populations to individual samples. A threshold value of 0.05 was established for *P* values < 0.05.

### 2.7. Sample Collection

Synovial tissue and peripheral blood samples from 5 patients of RA and 5 patients of OA were collected from the Nanxishan hospital of Guangxi Zhuang Autonomous Region. All subjects read and signed the informed consent form. The study was in conformance with the guidelines of the 1975 Declaration of Helsinki and was approved by the ethics committee of the Nanxishan hospital of Guangxi Zhuang Autonomous Region (2021NXSYYEC-001).

### 2.8. Quantitative Real-Time Polymerase Chain Reaction (qRT-PCR)

The total RNA was isolated from synovial tissue samples by using Trizol (Thermo, California, USA). After uniform quality between groups, total RNA was reverse transcribed to complementary DNA (cDNA) using PrimeScript™ RT Master Mix (TaKaRa, Tokyo, Japan). The qRT-PCR was performed using the SYBR Green Master Mix (Thermo, California, USA) using cDNA according to the manufacturer. The primer sequence of genes was shown in Table 1. Genes were normalized to GAPDH. Relative expression of mRNA was calculated through the 2^–*ΔΔ*CT^ method [33].

### 2.9. Western Blot

The synovial tissue samples of RA and OA were lysed on ice for 40 min in radio immunoprecipitation assay (RIPA) buffer (Beyotime, Shanghai, China). Proteins were loaded and separated by 10% SDS-polyacrylamide gel electrophoresis (SDS-PAGE) and then transferred onto polyvinylidene fluoride (PVDF) membranes. The membranes were incubated with the primary antibodies (all antibodies were purchased from Abcam) after blocking with skim milk. Protein bands were then incubated with corresponding secondary antibodies and detected by enhanced chemiluminescence (ECL) reagents. GAPDH protein was used as an internal reference protein.

### 2.10. Flow Cytometry

Peripheral blood samples were surface-labeled with anti-CD19 FITC (BD, California, USA), anti-CD3 PC5.5 (BD, California, USA), anti-CD8-PE antibody (BD, California, USA), or anti-CD45 PC7 (BD, California, USA) for 10 min at room temperature. The red blood cells in the blood were lysed with red blood cell lysate (BD, California, USA), then washed with PBS twice, and detected on the Dxflex Flow cytometry (Beckman, California USA). The results were analyzed using the Kaluza v2.1.1 software.

### 2.11. Statistical Analysis

Data analysis was used SPSS 20.0 software. Data were presented as mean ± standard deviations (SD) [34, 35]. Student's *t*-test was used to compare the differences between two groups [36]. The *P* value < 0.05 was considered statistically significant. Test level *α* = 0.05 (two-sided).

## 3. Results

### 3.1. The mRNA Signatures in Fibroblast of Synovial Tissue

**Figure 1:**
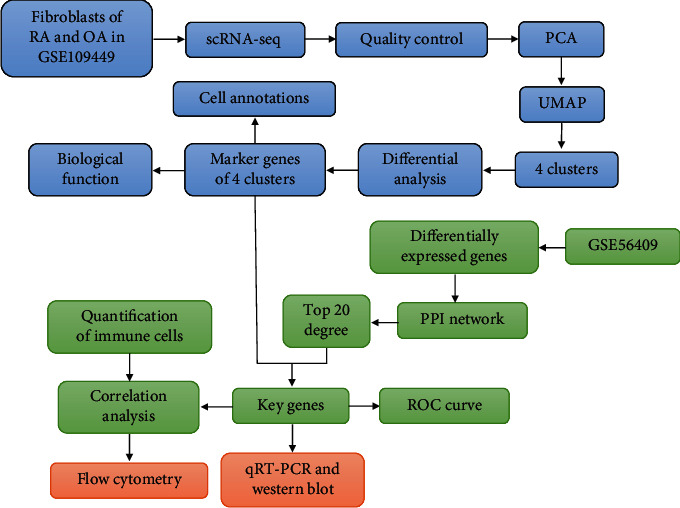
The flowchart of this study. Using single-cell sequencing data and transcriptome data to identify gene signatures and potential diagnostic markers of RA patient. OA: osteoarthritis; PCA: principal component analysis; PPI: protein-protein interaction; RA: rheumatoid arthritis; ROC: receiver operating characteristic curve; UMAP: uniform manifold approximation and projection.

**Figure 2:**
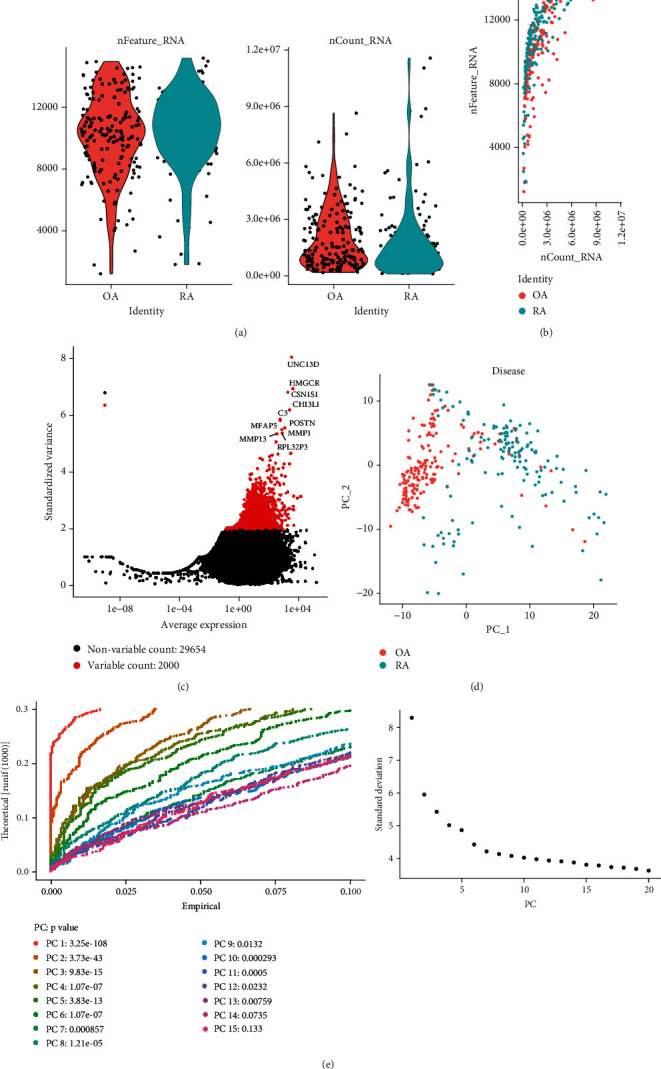
RA-related principal component genes were screened based on single-cell sequencing. (a) Quality control of synovial fibroblasts in RA. There were 31654 genes in 384 cells. RA: rheumatoid arthritis; OA: osteoarthritis. (b) The depth of sequencing was significantly correlated with the number of genes detected. Pearson's correlation coefficient was 0.68. (c) The variance diagram of gene expression for all fibroblast. The red dots represent 2000 highly variable genes, and the black dots represent nonvariable genes. (d) PCA demonstrates significant separations of cells between RA and OA. PC: principal components; RA: rheumatoid arthritis; OA: osteoarthritis. (e) PCA identified the 13 significant PCs. PC: principal components.

The article flow chart is shown in Figure 1. First, we analyzed scRNA-seq data from fibroblasts of 2 RA and 2 OA patients (GSE109449). Based on quality control and normalization of the data, 31654 genes were found in the 384 cells (Figure 2(a)). The number of detected genes was significantly correlated with sequencing depth (Figure 2(b)). Among 31654 corresponding genes, the variant analysis revealed 2000 highly variable genes (Figure 2(c)). In addition, principal component analysis (PCA) results showed significant separation between fibroblasts from RA and OA patients (Figure 2(d)). To identify the available dimensions and screen the related genes by PCA, we finally selected 13 principal components (PCs) to travel further analysis (*P* value < 0.05) (Figure 2(e)).

### 3.2. Cell Subpopulations in Fibroblast of RA and OA

**Figure 3:**
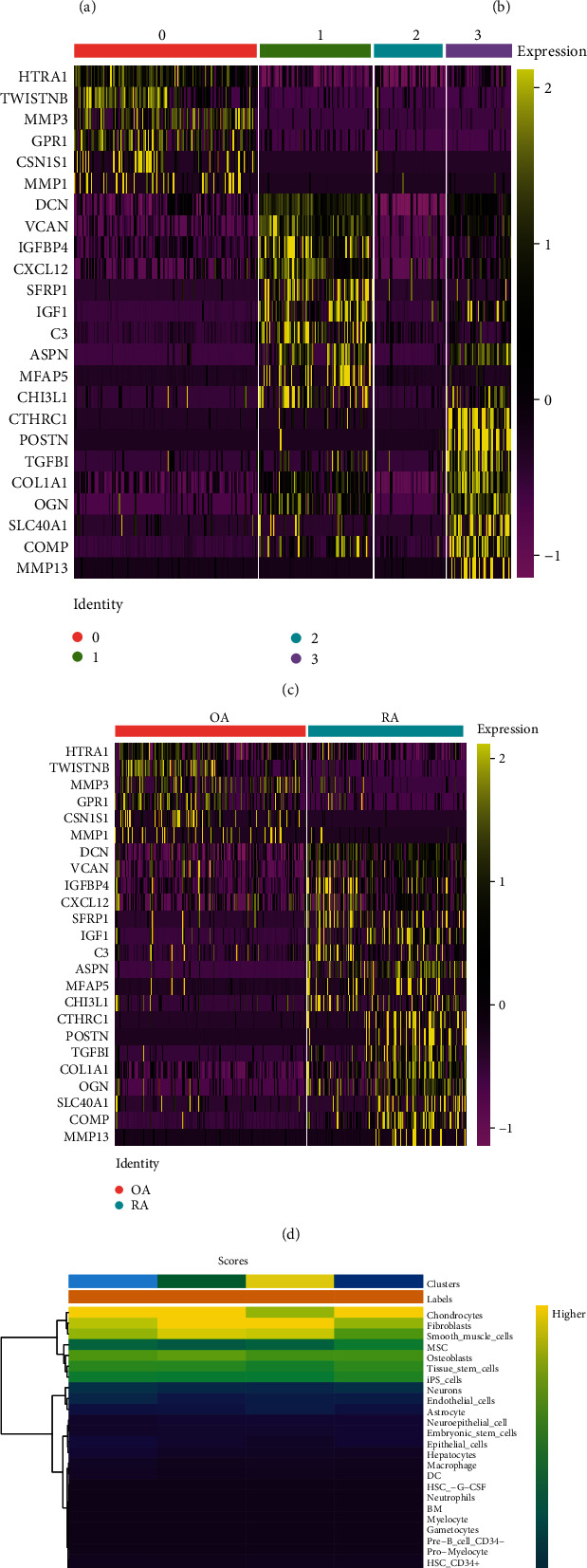
Identification of four cell subpopulations for fibroblast. (a) The UMAP algorithm reduced the dimensionality of 13 PCs and clustered into four cell clusters. UMAP: uniform manifold approximation and projection. (b) The cell clusters were matched to the sample types for RA or OA. RA: rheumatoid arthritis; OA: osteoarthritis; UMAP: uniform manifold approximation and projection. (c) Differential analysis identified 1561 gene markers. The top six gene markers for each cell cluster were listed on the left of heatmap. The colors from purple to yellow indicate the gene expression levels in each cell sample from low to high. (d) The expression of genes was matched to the sample types for RA or OA. RA: rheumatoid arthritis; OA: osteoarthritis. The colors from purple to yellow indicate the gene expression levels in each cell sample from low to high. (e) All clusters of cells were annotated according to the scores of singleR. The colors from blue to yellow indicate the gene expression levels from low to high.

To determine whether fibroblast subpopulations differ between RA and OA, we performed clustering analysis for the cells. Through the uniform manifold approximation and projection (UMAP) algorithm, we clustered fibroblasts into 4 separate clusters (Figure 3(a)). When these cell subpopulations were compared with the clinical phenotypes, we found that cluster 0 matched the OA group, and cluster 3 matched the RA group (Figure 3(b)). Next, we performed differential expression analysis, identifying 1561 marker genes in the four clusters (Figure 3(c)). Gene markers of cluster 0 were more highly expressed in OA than in RA, whereas gene markers of cluster 3 were more highly expressed in RA than in OA (Figure 3(d)). These clusters were annotated as cell types based on the score by singleR. However, different clusters were not annotated as different cell fibroblast subpopulations for RA and OA (Figure 3(e)).

### 3.3. Different Biological Function of Cell Subpopulations

**Figure 4:**
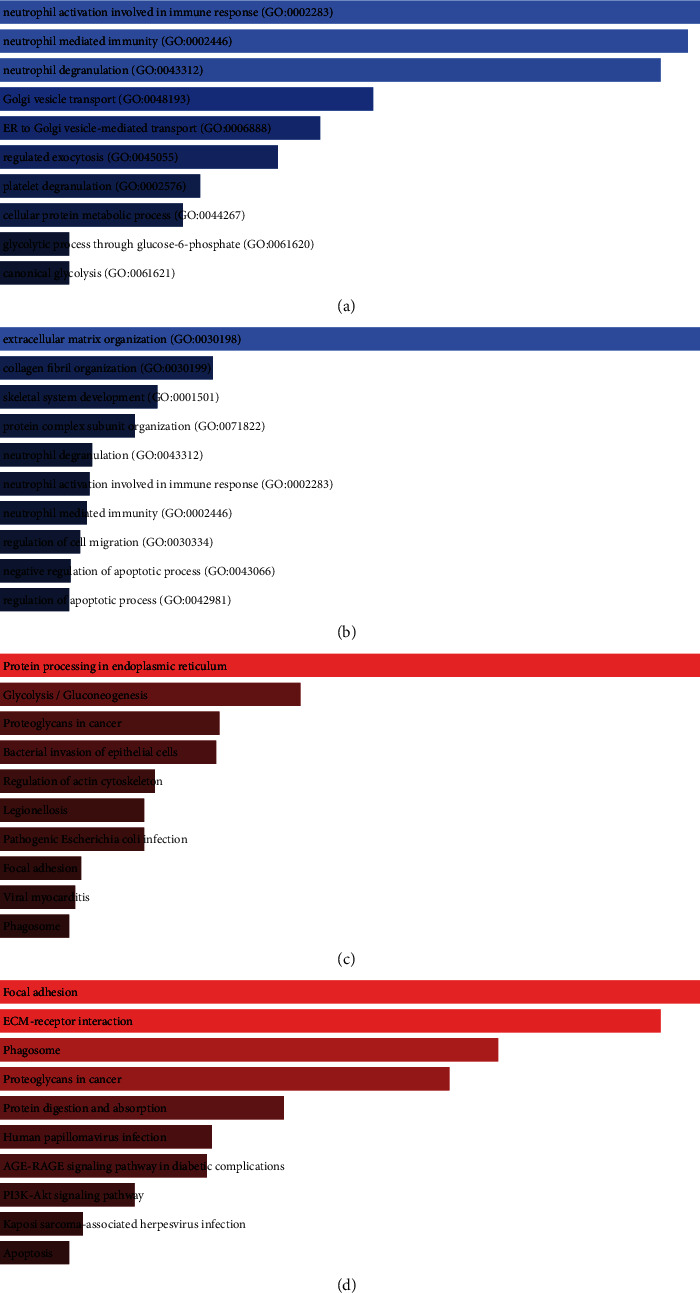
Enrichment analysis of gene markers of cell subpopulations matched to RA or OA. (a) Significantly enriched biological processes of cell subpopulations for cluster 0 who matched the OA patients. (b) Significantly enriched KEGG pathway of cell subpopulations for cluster 0. (c) Significantly enriched biological processes of cell subpopulations for cluster 3 who matched the RA patients. (d) Significantly enriched KEGG pathway of cell subpopulations for cluster 3.

To identify distinct biological roles for subpopulations of RA and OA patient fibroblasts, we performed enrichment analysis of gene markers of cluster 0 and cluster 3. It was found that gene markers of cluster 0 were significantly enriched in biological process (BP) of neutrophil activation involved in immune response, neutrophil-mediated immunity, and neutrophil degranulation (Figure 4(a)). Gene markers of cluster 3 were significantly enriched in BP of extracellular matrix organization, collagen fibril organization, and skeletal system development (Figure 4(b)). KEGG enrichment results showed that gene markers of cluster 0 were significantly enriched in protein processing in protein processing in the endoplasmic reticulum, glycolysis/gluconeogenesis, and proteoglycans in cancer (Figure 4(c)). While gene markers of cluster 3 were significantly enriched in focal adhesion, ECM receptor interaction, and phagosome (Figure 4(d)).

### 3.4. Gene Expression in Fibroblasts of RA

**Figure 5:**
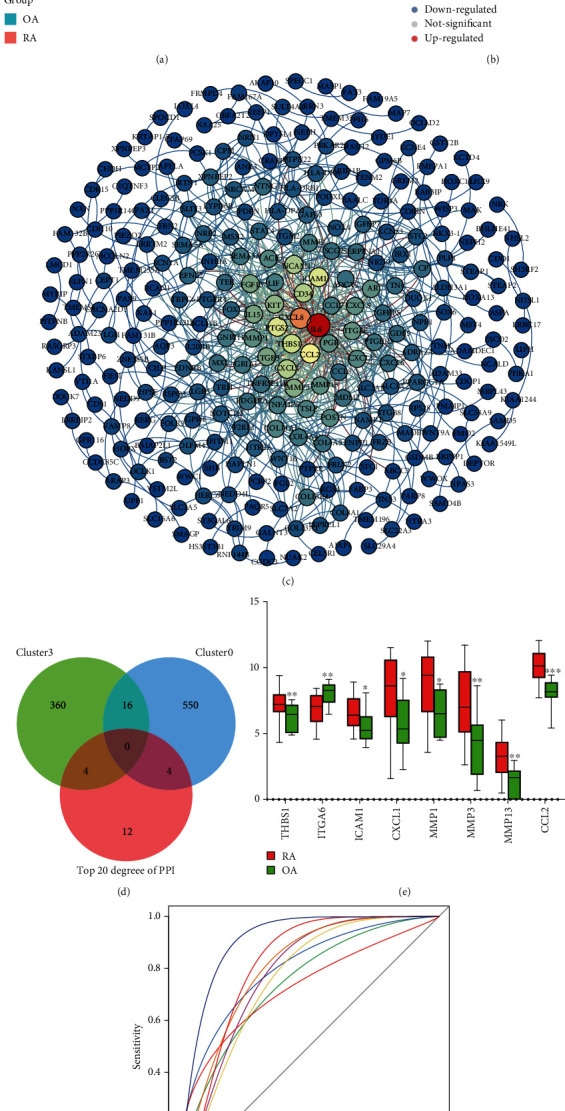
Identification of differentially expressed genes between RA and OA in GSE56409. (a) Heatmap of differential gene expression in RA and OA. RA: rheumatoid arthritis; OA: osteoarthritis. Red are upregulation and blue are downregulation. (b) Volcano plot of differentially expressed genes between RA and OA. Red are upregulation and blue are downregulation. (c) PPI network of differentially expressed genes. The colors from blue to red, representing the greater degree to which genes are connected in the network. (d) Venn diagram of the intersection among cluster 0, cluster 3, and the top 20 degrees in the PPI network. Then obtained eight key genes. (e) Differential expression of key genes between RA and OA in GSE56409. RA: rheumatoid arthritis; OA: osteoarthritis. (f) ROC curve of key genes for predicting diagnosis of RA. AUC: area under ROC curve.

Afterwards, we obtained 478 differentially expressed genes (DEGs) using gene expression data in fibroblasts from RA patients and OA patients (Figure 5(a), Table S1). The 294 upregulated DEGs and 184 downregulated DEGs were included (Figure 5(b)). The PPI network of DEGs with interactions was acquired through a string database (Figure 5(c)). We screened the top 20 greatest degree genes of connection in the PPI network as candidates (Table 2). Comparing with the gene markers of clusters, we found that MMP3, ITGA6, MMP1, and CXCL1were the intersection genes for cluster 0, and THBS1, CCL2, MMP13, and ICAM1 were the intersection genes for cluster 3 (Figure 5(d)). Therefore, we considered that these 8 genes might be associated with arthritis and were defined as key genes. THBS1, CCL2, MMP13, and ICAM1 may be potential markers for RA. Among the differential results, THBS1, CCL2, MMP13, ICAM1, MMP3, MMP1, and CXCL1 showed higher expression in RA compared with OA, while ITGA6 showed lower expression in RA (Figure 5(e)). Receiver operating characteristic (ROC) curve results showed that CCL2 and MMP13 had a good predictive diagnostic role for RA (Figure 5(f)). CCL2 and MMP13 with the highest area under the receiver operating characteristic curve (AUC) values (AUC > 0.8).

### 3.5. Immune Cell Changes in RA Patients

**Figure 6:**
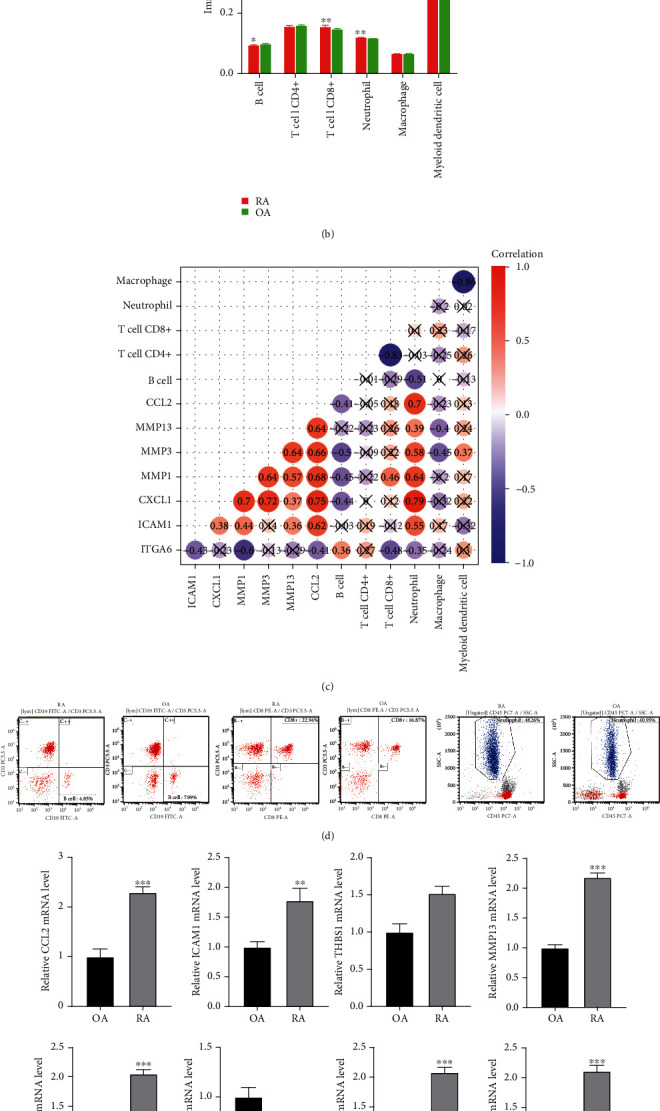
Changes of immune cell and key genes in RA patients. (a) Infiltration levels of immune cells in RA and OA. RA: rheumatoid arthritis; OA: osteoarthritis. Red represents high infiltration and blue represents low infiltration. (b) The difference of infiltration for immune cells in RA compared to OA. RA: rheumatoid arthritis; OA: osteoarthritis. (c) Correlations between immune cells and key genes were analyzed through Pearson correlation. Node color from blue to red represents negative to positive correlation. ×*P* > 0.05. (d) The levels of CD8+T cell, neutrophils, and B cell in blood samples of RA and OA patients were detected by flow cytometry. (e) QRT-PCR was used to detect the mRNA levels of key genes in synovial tissue of RA and OA patients. ^∗∗^*P* < 0.01, ^∗∗∗^*P* < 0.001. (f) Western blot was used to detect the expression of key genes in synovial tissue of RA and OA patients.

Immunoinflammation appeared in our enrichment results, especially cluster 0 which matched OA. To compare the differences in immune responses between RA and OA patients, we quantified the infiltration of immune cells according to the immune score (Figure 6(a)). We found that B cells were significantly decreased, and CD8+T cells and neutrophil were significantly increased in RA compared to OA (Figure 6(b)). The results of the correlation analysis with the key genes showed that neutrophil was significantly associated with all of the key genes (Figure 6(c)).

Importantly, we validated significant results of our analysis in blood or synovial tissue samples from RA and OA patients. Using flow cytometry, we found that the levels of CD8+T cells and neutrophil were significantly higher in RA patients than in OA patients, while the levels of B cells were significantly decreased (Figure 6(d)). QRT-PCR results found that the mRNA levels of THBS1, CCL2, MMP13, ICAM1, MMP3, MMP1, and CXCL1 were higher in RA compared with OA, and ITGA6 was lower expressed in RA (Figure 6(e)). The differential expression results of the genes were also validated by Western blot experiments, except ITGA6 (Figure 6(f)).

## 4. Discussion

Previous studies have highlighted fibroblasts as potential therapeutic targets for RA [37]. In the present study, we sought to identify cell subpopulations of RA patient fibroblasts by comparing the results of single-cell sequencing of synovial tissue fibroblasts from RA and OA patients. And describe the contribution of different cell subpopulations to the molecular mechanisms of RA. Transcriptome data were further combined to screen for potential fibroblast-specific markers. Importantly, we utilized molecular experiments to validate key results. These are particularly important, as such biomarkers may contribute to the early diagnosis and early treatment of the disease.

Unfortunately, we did not get different annotations for the different cell subpopulations. Of the four subpopulations identified, cluster 0 may be more representative for OA, whereas cluster 3 may be representative for RA. Gene markers for cluster 3 (MMP13, COMP, SLC40A1, OGN, COL1A1, and TGFBI) play a significant role in collagen, fibronectin, and laminin interactions that increase fibroblast migration, invasion, and cell adhesion [38–40]. In healthy joints, synovial fibroblasts form a layer in synovial tissue with a thickness of one to two cells [41]. In synovial tissue of RA patients, synovial fibroblasts form thicker layers (15-20 cells thick), mainly due to a higher proliferation rate, and the formation of antiapoptotic properties [42]. In addition, these gene markers are also major drivers of the inflammatory response in RA patients and are identified as potential markers for RA [5, 43]. The synovium is a major target of inflammation in RA.

Here, we first recognize that distinct fibroblast subpopulations differ in their molecular functions. Gene markers for cluster 0 are mainly enriched in neutrophil-mediated immunity, glycolysis/gluconeogenesis. Aberrant neutrophil responses contribute to tissue damage and are associated with arthritic pathological conditions [44, 45]. Aerobic glycolysis is manifested by inflammatory signals or rapid cell division, reflecting systemic inflammation [46]. Marker genes of cluster 3 were mainly associated with ECM receptor interaction, collagen fibril organization. Activated synovial fibroblasts produce multiple ECM remodelling components, such as matrix metalloproteinases, cytokines, and chemokines, which actively promote cellular resorption and infiltration of the joint, perpetuating and perpetuating joint inflammation [47]. Previous studies have found that collagen fibril organization is associated with the pathology of RA [48]. The presence of thinner fibers and high concentrations of collagen cleavage products have been associated with RA events [49].

Among the key genes we identified, chemokine ligand 2 (CCL2) and matrix metalloproteinases 13 (MMP13) were predicted as potential diagnostic markers for RA. Essential cytokines in the development of RA are IL-6, and IL-6 activation of endothelial cells increases adhesion molecule expression and CCL2 production [50]. CCL2 levels are increased in the plasma and synovial fluid of RA patients, closely correlating with increased joint infiltration of immune cells, particularly macrophages [51]. Studies have shown that CCL 2 is an effective therapeutic target for RA patients [52, 53]. MMP13 expression is increased in synovial fibroblasts of RA patients [54]. Elevated expression of MMP13 in RA patients may promote fibroblast migration and invasion [55]. MMP13 is also an effective therapeutic target for multiple drugs in RA patients [56]. In contrast to OA patients, we observed upregulation of CCL2, MMP13 in RA patients, suggesting potential novel targets.

Specifically, in the differential immune cell infiltration results, we found that the levels of CD8+T cells and neutrophils were higher in RA fibroblasts than in OA patients, whereas the levels of B cells were decreased. CD8+T cells are activated in RA and produce a large number of chemokines and proinflammatory cytokines [57, 58]. Neutrophil entry into the synovium is an important feature of the RA inflammatory response, which is again fueled by an intricate network of cytokines [59]. Neutrophils damage cartilage in synovial fluid and damage surrounding tissues, leading to a state of oxidative stress resulting from the release of reactive oxygen species (ROS) and increasing inflammatory conditions [60]. The number of total B cells in the blood of rheumatoid arthritis patients has been shown to be reduced compared to healthy controls [61]. However, it has also been shown that B cell depletion in RA patients is a potential therapeutic intervention [62].

Some limitations are included in this study. The low number of samples we analyzed may have biased the interpretation of the results. Whether the identified potential targets have clinically significant will requires subsequent in-depth exploration. Although we validated the differences in key genes and immune cells between RA and OA, this has some limitations for interpretation of validation results as we failed to isolate fibroblast samples for experimentation.

## 5. Conclusion

Chemokine and matrix metalloproteinases (MMPs) signaling plays an important role in RA pathogenesis, as several chemokines and their receptors have been implicated in the inflammatory response and immune infiltration in fibroblasts. Therefore, targeting chemokines and MMPs is a suitable approach for the diagnosis and treatment of RA, especially CCL2, and MMP13. The significance of potential target genes in RA disease is evaluated herein. This information provides a solid background for the development of new drugs or other treatments.

## Figures and Tables

**Table 1:** The primers of this study.

Genes	Primers
GAPDH	F: 5′-TGACCGTCGGAGTCAGGGATTT-3′
	R: 5′-GCCAACGAATTTGCCATGGGTGG-3′
ICAM1	F: 5′-TGCAAGAAGATAGCCAACCAAT-3′
	R: 5′-GTACACGGTGAGGAAGGTTTTA-3′
CXCL1	F: 5′-AAGAACATCCAAAGTGTGAACG-3′
	R: 5′-CACTGTTCAGCATCTTTTCGAT-3′
MMP1	F: 5′-AGATTCTACATGCGCACAAATC-3′
	R: 5′-CCTTTGAAAAACCGGACTTCAT-3′
ITGA6	F: 5′-GTGCTTGCTCTACCTGTCGG-3′
	R: 5′-GCTCCCGGGGTCTCCATATT-3′
MMP3	F: 5′-GGGTCTCTTTCACTCAGCCAACAC-3′
	R: 5′-ACAGGCGGAACCGAGTCAGG-3′
CCL2	F: 5′-ACCAGCAGCAAGTGTCCCAAAG-3′
	R: 5′-TTTGCTTGTCCAGGTGGTCCATG-3′
THBS1	F: 5′-TTTGACATCTTTGAACTCACCG-3′
	R: 5′-AGAAGGAGGAAACCCTTTTCTG-3′
MMP13	F: 5′-CACTTTATGCTTCCTGATGACG-3′
	R: 5′-TCTGGCGTTTTTGGATGTTTAG-3′

**Table 2:** Top 20 genes with the largest degree in the PPI network.

Genes	Degree
IL6	60
CXCL8	40
CCL2	30
PTGS2	28
ICAM1	28
THBS1	27
CXCL1	25
CD34	25
NCAM1	24
KIT	22
MMP3	21
ITGB3	20
IL15	20
FGF13	20
ITGA6	18
MMP13	17
PGR	17
AR	17
CXCL9	17
MMP1	17

## Data Availability

The raw data can be found in GSE109449 and GSE56409.

## Supplementary Materials

Supplementary Materials Table S1: the differentially expressed genes between RA patients and OA patients in GSE56409.
